# Review of the twelfth West Coast retrovirus meeting

**DOI:** 10.1186/1742-4690-2-72

**Published:** 2005-11-17

**Authors:** Sheila M Barry, Marta Melar, Philippe Gallay, Thomas J Hope

**Affiliations:** 1 Department of Cell and Molecular Biology, College of Medicine, Northwestern University, Chicago, IL 60611, USA; 2 Department of Microbiology and Immunology, College of Medicine, University of Illinois at Chicago, Chicago, IL 60612, USA; 3 The Scripps Research Institute, La Jolla, CA 92037, USA

## Abstract

Every year the Cancer Research Institute from University of California at Irvine organizes the West Coast Retrovirus Meeting where participants have a chance to discuss the latest progress in understanding the pathology of retroviruses. The 12^th ^meeting was held at the Hyatt Regency Suites in Palm Springs, California from October 6^th ^to October 9^th ^2005, with the major focus on human immunodeficiency virus (HIV) pathogenesis. Philippe Gallay from The Scripps Research Institute and Thomas J. Hope from Northwestern University organized the meeting, which covered all the steps involved in the lifecycle of retroviruses with an emphasis on virus:host interactions. The trend in research appeared to be on the restriction of viral infection, both by the endogenous, cellular restriction factors, as well as by the potential antimicrobial compounds of known or unknown mechanisms. Additionally, new stories on the inevitable feedback from the host immune system were presented as well. HIV still represents a challenge that an army of motivated people has been working on for over 20 years. And yet, the field has not reached the plateau in knowledge nor enthusiasm, which was proven again in October 2005 in Palm Springs.

## Review

### Viral Entry

John Young of the Salk Institute began this session by describing work his lab has recently completed in understanding cellular requirements for replication of Murine Leukemia Virus (MLV) [1]. Through use of chemically mutagenized CHO cells, they identified five clones that became resistant to MLV infection. Additional studies revealed this restriction was specific to the MLV core. After confirming the virus was blocked prior to integration, the clones were separated into two phenotypes, those which blocked reverse transcription early and those which allowed reverse transcription and nuclear entry, but prevented viral integration. Young and colleagues are currently identifying cellular factors involved in the latter phenotype. While the exact identities of these cellular factors were not revealed, Young shared that they believe one is an enzyme and the other a putative transcription factor.

Pankaj Kumar from Lorraine Albritton's lab at the University of Tennessee continued this theme by examining cellular factors involved in Moloney MLV entry. Previous work found that the exposure of MLV to proteases enhanced the viral infectivity and certain cell lines, including XC cells, innately possessed proteases that could facilitate MLV infection. The group decided to focus on cathepsins, since expression of these cellular proteases is induced under these conditions. They found a broad spectrum cathepsin inhibitor as well as a cathepsin B-specific inhibitor reduced Moloney MLV infectivity. Additionally, treatment of viral particles with cathepsin B resulted in cleavage of the surface glycoprotein (SU). They postulate Moloney MLV encounters cathepsin B within early lysosomes and the ensuing cleavage of SU facilitates fusion and entry steps.

Two talks turned attention to the involvement of HIV envelope glycoprotein gp41 in early steps of viral infection. In work previously published by his lab, John Day of the University of California San Diego and others determined the membrane proximal tyrosine based sorting signal of gp41, Y712xxL, was important in viral entry and infectivity and was involved in virion incorporation of the envelope glycoprotein (Env) only in some cell lines [2]. They hypothesized this enhancement of viral infectivity resulted from the virus using adaptor protein complexes to traffic Env to specific cellular membranes. Gp41 has few motifs that are known to interact with adaptor proteins (AP); Y712xxL interacts with AP-2, while the C-terminal double leucine motif (LL855/856) binds to AP-1. Thus, both signals were evaluated for their ability to affect intracellular localization and viral infectivity. In studies using CXCR4 tropic HIV-1, LL855/856 was found to have no effect on viral infectivity or entry, a sharp contrast from the observed viral dependence on Y712xxL. However, no difference was observed in intracellular localization of either mutant compared to wildtype. This suggests the Env sorting signals may not be involved in targeting viral morphogenesis to specific cellular membranes. Interestingly, when these signals were evaluated with CCR5 tropic HIV-1, neither the LL855/86 nor the Y712xxxL sorting signal had any effect on viral infectivity. This observation indicated the significance of the tyrosine-sorting signal in viral infectivity is dependent on the tropism of the HIV Env ectodomain.

Michael Kay from the University of Utah presented his lab's efforts in understanding the ineffectiveness of vaccine development against the N trimer of gp41. Following gp120 binding to coreceptor, gp41 undergoes conformational changes, from a pre-hairpin state where both N and C peptides are exposed, to the formation of a six-helix bundle, where a trimer of N peptides (N trimer) is surrounded by three C peptides. Within this N-trimer is a highly conserved pocket which has become the target of most vaccine development. Unfortunately, little progress has been made in creating an effective anti N trimer vaccine. Kay and collaborators considered a potential obstacle to vaccine development was the accessibility of the region to bulky inhibiting proteins. To evaluate this possibility, this group used a C-peptide inhibitor that was attached via a flexible linker to several cargo proteins of various sizes. They found the potency of this inhibitor decreased with increasing cargo protein size. Increasing the length of the flexible linker region could restore potency [3]. This suggests a severe steric block in gp41 to neutralizing antibodies.

The session ended with a talk by Marta Melar from Thomas Hope's lab at Northwestern University on coreceptor dependent signaling during HIV entry. By measuring changes in intracellular calcium (Ca^2+^) levels as a marker for signaling through coreceptor, Melar observed that signaling was coreceptor specific, responsive to both monomeric and virion bound gp120, and dependent on CD4. The fluorescent microscopic technique employed in these studies allowed Melar to quantate the number of virions bound to Ca^2+^- fluxing cells. An average of four virions was determined to be sufficient for Ca^2+ ^mobilization in primary unstimulated CD4^+ ^T cells.

### Vif, Vpr and Nef

Several interesting talks emphasized the ability of these accessory HIV proteins to evade the host immune system in order to make a perfect niche for viral replication in the hostile target cells. The stories on Vif protein focused on its ability to protect the virions from incorporation of the cellular apolipoprotein B mRNA-editing enzyme-catalytic polypeptide-like-3G (APOBEC-3G or A3G) [4]. A3G has cytidine deamination activity and can use newly reverse transcribed viral genome as a substrate, leading to the loss of viral fitness through introduction of G-to-A hypermutations in the plus strand of the cDNA

Jason Kreisberg, a graduate student from Warner Greene's lab from University of California at San Francisco, presented the ongoing work in the lab regarding the mechanism of A3G dependant HIV restriction in secondary lymphoid organs. This work is the continuation of already published data [5] on two existing forms of A3G, high and low molecular weight A3G, where only a low molecular weight form exhibits enzymatic activity. RNase treatment was shown to facilitate the switch from the high into low molecular weight form. Kreisberg emphasized the correlation between the presence of the A3G molecular weight form and permissiveness of the cell type to infection. They found resting peripheral CD4^+ ^T cells that are not permissive for infection express the enzymatically active version of A3G. However, when isolated from tonsils and cultured in conditioned media, this cell type becomes permissive to HIV infection. Cytokines, specifically IL-2 and IL-15, may have a role in this *in vivo *switch from low to high molecular weight A3G. These data could shed some light on the role of the target cell A3G opposing the well-established mutagenizing role of A3G on the HIV genome in the producer cells. Since only high molecular weight A3G is incorporated into ΔVif virions, it was not known how A3G gains its activity in the target cells. The virally encoded enzyme RNaseH may be doing the virus a contra-favor, by functioning as the facilitator of A3G cytidine deaminase activation.

The session continued with another keynote lecture given by Nathaniel Landau from the Salk Institute for Biological Studies from San Diego. This talk was focused on the species-specificity of the Vif:A3G interaction. The ability of Vif to block the antiviral activity of A3G is species-specific [6], where the positive charge of single Asp in the position 128 within human A3G is responsible for recognition of HIV Vif and its interaction. From mutational analyses, Landau and his collaborators found that out of two active sites within APOBEC family of enzymes, the first active site (AS1) plays a role in encapsidation of the enzyme into the ΔVif virions, where AS2 is responsible for deamination of the substrate, the negative DNA single strands in a newly synthesized viral genome. As well, the group found a graded deamination frequency, from low at the 5'-end to higher towards the 3'-end, most likely a phenomenon affected by the mechanism of the reverse transcription reaction and the availability of negative strand cDNA to the A3G-induced mutation.

The following talk from Michael Emerman’s group at the Fred Hutchinson Cancer Research Center continued the discussion on different aspects of antiviral properties of APOBEC enzymes.  Shari Kaiser addressed the question if the uracil DNA glycosylase 2 (UNG2) is involved in the antiviral effects of A3G.  Previously, this enzyme was postulated to work one step downstream of A3G, enabling G-to-A hypermutations to occur.   However, Kaiser found that virus replication in either target or producer cells was not affected as compared to the positive control in either ung-/- cell line or after the UNG2 inhibitor treatment in the producer cells.  This implied UNG2 was dispensable for the fitness of the virus contrasting with a recent publication [7].

Another focus on host:virus interaction came from Morehouse School of Medicine in Atlanta, where Michael Powell's group works on HIV infectivity enhancement through the direct Nef and CypA interaction. This work was based on the hypothesis that CypA acts as a linker between HIV Nef and the viral core, interacting with Nef at its N-terminus and the core through its C-terminus. They speculate this interaction between Nef and CypA can facilitate the uncoating process in the target cells, since induction of natural endogenous reverse transcription (NERT) in intact virions could overcome the lack of either protein. They also showed a Nef:CypA fusion protein, which efficiently got incorporated into virions, restored infectivity of ΔNef virions. Interestingly, the group also suggested that the ability of SIV Nef to bind core directly might mask the restriction effect of cellular restriction factor TRIM5α that is known to interact with viral core, since HIV virions expressing SIV Nef were able to bypass the restriction point of simian TRIM5α and replicate in simian MAGI cells. That was also the case with NERT induced wild type HIV in simian MAGI cells.

The mechanism of MHC class II invariant chain (Ii) up-regulation was another Nef function discussed during this session. Richard Mitchell from University of California at San Diego presented work on the importance of the di-leucine sorting motif E_160_xxxLL found at the C-terminus of HIV Nef and its potential role in providing a sorting endocytic signal for down-regulation of the surface expression of CD4, coreceptors CXCR4 and CCR5, MHC I and II and up-regulation of MHC II-Ii complexes at the cell surface. By using yeast three-hybrid system and GST-pulldown assays, the group found that residues E_160 _and LL were important for up-regulation of the surface Ii expression. This is another report explaining the role of this accessory HIV protein in enhancing the infectivity of the virus, by altering the immune response of the host.

### Uncoating and budding

The next panel began with two keynote lectures, both focusing on the issue of viral restriction in different hosts. Jaquelin Dudley from University of Austin, Texas, introduced us to the world of mouse resistance to multiple pathogens. Her group observed that certain strains of inbred mice carry an endogenous mouse mammary tumor virus (MMTV) that is replication deficient but does express the virally encoded superantigens (Sags). Sags expression results in a depletion of specific T cell subsets. These mice, when infected with exogenous MMTV, are prone to the development of mammary gland tumors. The group created MMTV-negative mice, which were found to be protected from a replication-competent, exogenous MMTV, type B leukemogenic virus and *Vibrio cholerae*. Subsequently infected with MMTV, MMTV-null mice lacked an immune response to the virus and lacked the tumor development. Genetic analysis revealed that the susceptibility to MMTV infection of endogenously infected mice was a recessive feature and that a single MMTV gene product was rendering these animals susceptible to infection, implying a novel mechanism of resistance to both viral and bacterial pathogens.

Another story on resistance to viral infection dealt with HIV restriction in Old World monkeys by a cellular restriction factor named TRIM5α. This molecule is a big hit in HIV research, ever since the Sodroski group from Harvard University published data from a primary rhesus monkey lung fibroblasts cDNA library screen for the resistance to HIV-1 infection [8]. Matt Stremlau gave us an insight on how this restriction factor might work in order to block the incoming virus at the post-entry step but pre-integration. Previously defined interaction of TRIM5α with the viral core served as a starting point to speculate that TRIM5α could either stabilize the capsid core, cause rapid disassembly of the core or target the capsid (CA) for proteasomal degradation. All three outcomes could have an impact on the very time-sensitive process of the reverse transcription. From their work on *in vitro *assembled HIV cores, representing highly ordered tubular structure of p24 CA hexamers [9], the group found that TRIM5α in its functional trimeric form binds only to the core composed out of CA hexamers, but does not bind to p24 CA monomers. Since their data indicate that the proteasomal inhibitors did not recover the loss of the oligomeric into the monomeric CA form, the group speculated that TRIM5α most probably acts to rapidly disassemble the core and that would impair the reverse transcription process, also implying the species-specific blocking mechanism on the conformational level.

On the other hand, Philippe Gallay from The Scripps Research Institute showed recent data arguing that HIV CA but not the matrix protein was being targeted for degradation, although other than through proteasomal pathway, since proteasomal inhibitors did not fully rescue the RhMTRIM5α mediated degradation of the HIV CA. This group argued that TRIM5α restriction occurs at the level of accelerated degradation of the core, possibly also affecting the nuclear import of the preintegration complex.

Microscopy based approach to study the cellular localization of TRIM5α in living cells came from Thomas Hope group at Northwestern University. The audience had a chance to see that both exogenous and endogenous TRIM5α formed cytoplasmic bodies, but the proteins were also found in the nuclei. The cytoplasmic bodies are highly dynamic hollow structures and their formation is speculated to be relevant in the TRIM5α function as a restriction factor. The morphology of the bodies could be altered with the proteasome inhibitor MG132, where the smaller bodies merged to form bigger structures. The group is currently investigating the effect of MG132 on the TRIM5α restriction potency.

An interesting study came from Bruce Torbett's group, where Christina Swan presented work on the design of HIV based vectors for gene therapy in human stem and T cells based on the HIV tropism. However, since monkeys would be the animal model for the vector design trials, the problem of the intrinsic cellular restriction of incoming HIV virions by the RhTRIM5α arose. In order to overcome this restriction problem, the group decided to test numerous HIV CA mutants and found that incorporation of the naturally occurring four amino acid substitutions in the CypA binding site of HIV Gag/Pol allowed for the restriction escape and therefore higher transduction efficiencies in primary human and monkey cell lines. These mutations allowed independence of CypA in human cells and loss of TRIM5α recognition because of the lack of CypA incorporation into the virions in monkey cells.

Another way to block HIV infection besides engaging the endogenous restriction machinery is to test the potential antimicrobial compounds. Christopher Aiken from Vanderbilt University introduced us to the HIV-1 maturation inhibitor 3-*O*-{3',3'- dimethylsuccinyl}-betulinic acid (DSB). DSB specifically inhibits HIV replication by delaying the last step in the Gag maturation: the release of the spacer peptide SP1 from the C-terminus of CA. However, the inhibitory effect was not due to the protease (PR) inhibition, since PR inactivation stabilized the DSB:CA complex. The escape mutants in CA-SP1 junction were not incorporating DSB and were now rendered resistant to it. Moreover, Aiken showed data supporting the hypothesis that DSB binds to a pocket formed by Gag oligomerization, an interaction that sterically inhibits PR from binding [10]. The compound had to be present at the time of the viral assembly in order to inhibit the viral replication in a dose-dependant manner and was also shown to be a weak fusion inhibitor.

The session on viral uncoating and budding was concluded by the talk from Wesley Sundquist's group from University of Utah. Their research focuses on structural proteomics to understand the process of ubiquitinated Gag recognition by the cellular sorting machinery through endosomal sorting complexes required for transport (ESCRT I-III), utilization of multivesicular bodies formation and the energy of ATP hydrolysis in the viral protein sorting, assembly and budding. Melissa Stuchell-Brereton presented recently published data on the latest structural analysis of one of the players in this cellular machinery that mediates recycling of the sorting apparatus from the cargo, namely VPS4A AAA ATPase [11]. Stuchell-Brereton described the novel three-dimensional structure of VPS4A C-terminal helix and N-terminal fragment: a microtubule interacting and transport domain (MIT). Data suggested that the VPS4A MIT domain directly binds the C-terminus of one of the ESCRT-III proteins, allowing the formation of the ring structure, where VPS4 proteins may serve to unfold, translocate and therefore recycle the members of ESCRT-III family through the ring pore, indirectly facilitating HIV budding.

### HIV Inhibition and Activation

David Margolis of the University of North Carolina at Chapel Hill gave the keynote lecture of this session, recapping work his lab has completed in depleting latent HIV infection from resting CD4^+ ^cells [12]. In the twenty years since the discovery of HIV, several anti-retroviral therapies have been attempted, many of which have terrible side effects and are not well tolerated by patients. In addition, while viremia may be reduced during treatment, viral load increases significantly once therapy is stopped. A major obstacle to eradication of HIV infection is the persistence of a latent viral reservoir within resting CD4^+ ^cells. Therefore, stimulating HIV expression from these resting CD4^+ ^T cells would allow the immune system to recognize infected cells and target the infection more efficiently. Histone deacetylase 1 (HDAC1) is instrumental in maintaining latency of integrated HIV, thus inhibitors of HDAC1, such as the FDA-approved valproic acid (VPA), may assist in expression of HIV from resting CD4^+ ^cells. To examine this hypothesis, Margolis' group supplemented the treatment of four patients with therapeutic doses of VPA. Infection of CD4^+ ^cells decreased in all patients, with three exceeding expectations. While considerable work still remains to be completed, these results suggest VPA may be a promising addition to HIV treatment.

The subsequent two talks examined the participation of certain transcription factors in HIV expression. Jonathan Karn from Case School of Medicine and his lab have recently completed research studying the molecular mechanisms of NF-κB and other transcription factors in expression of integrated HIV. To conduct these studies, they created a population of T cells that possessed stably integrated proviral HIV genomes that encoded GFP. The group used these cells to evaluate the activation of HIV transcription, as they turn green following treatment with TNF-α. Additionally, they were able to evaluate the distribution of RNA polymerase (RNA pol) II along HIV LTR as well as the kinetics of proviral activation following recruitment of TFIIH and NF-κB to the promoter and provirus by using chromatin immunoprecipitation (ChIP). These studies revealed recruitment of NF-κB coincided with an accumulation of RNA pol and TFIIH within the nucleus. Interestingly, induction of transcription was found to be transient, with levels of RNA pol, TFIIH, and NF-κB returning to pretreatment levels within 90 minutes following activation, only to increase in a second cycle of induction 3 to 5 hours later. Although the mechanism is more complicated, T cells stimulated though the T cell receptor CD3 experienced a similar trend. Initially, NFAT was observed to be selectively mobilized, only to be replaced by NF-κB within 30 minutes. These observations suggest the induction of HIV transcription is a multifactorial process that is cyclical in nature, not the sustained event as previously supposed.

Andrew Rice from Baylor College of Medicine presented his lab's investigation of the role of 7SK small nuclear RNA (7SK) in P-TEFb function and, in doing so, challenged the previously described model for these proteins in HIV expression [13]. P-TEFb is a RNA pol II transcription factor that is composed of Cdk9 and cyclin T1, T2 or K. The HIV Tat protein targets the Cdk9/cyclin T1 P-TEFb to activate transcription of the viral genome. Much of this P-TEFb is complexed to 7SK and HEXIM proteins, however, and this complex has been demonstrated to have decreased kinase activity *in vitro*. Rice and colleagues examined 7SK and HEXIM in primary cells and found expression of these proteins positively correlated with the activation state of the cells. Additionally, there was no observed difference in expression of endogenous genes or integrated HIV provirus when siRNA was used to deplete 7SK, although expression of reporter plasmids increased. Another interesting observation was that apoptosis was induced within 72 hours in 7SK depleted cells. This group postulates these findings indicate 7SK plays a significant role in P-TEFb function, one that merits further investigation.

Wendong Yu from Baylor College of Medicine at Houston, Texas discussed the function of cyclin T1 in Mono-Mac-6 (MM6) cells as a model for primary monocytes-to-macrophages differentiation. The work was based on the observation that the differentiation of monocytes into macrophages (MΦs) is followed by the increasing levels of CycT1, which together with CDK9 constitutes for P-TEFb, a factor necessary for Tat-induced transcriptional activation. In the early MΦs, both CycT1 and Tat levels were elevated, but there was a significant loss of CycT1 expression in late MΦs that could be restored with PMA, IFNγ or LPS induced signaling. Indeed, when CycT1 was knocked out in MM6 cells using a shRNA approach, microarray analysis revealed downregulation of ~13% genes, where ~11% genes were PMA-inducible ones. This data emphasized the role of the CycT1 induction in MΦ differentiation and upregulation of ~11% genes.

Mary Lewinski from Bushman's group gave us an insight into the integration target specificity of HIV and MLV [14]. After extensive integration site cloning, mapping to the genome and considerable statistical analyses, the group concluded that the chromosomal environment influences the expression of integrated sequences and that different retroviruses show disparate preferences for integration of their genome into the host chromosomes. To understand which viral proteins orchestrate the choice for the integration location within the host genome, numerous chimeric viruses between MLV and HIV were tested for the preferential sites for integration. The interesting conclusion was that not one, but a pair of genes, Gag and integrase, worked synergistically to determine the integration site specificity.

The last two talks in this session were reserved for potential antiviral agents. The talk from Vanderbilt University by Derya Unutmaz focused on VacA toxin, produced by bacterium *Helicobacter pylori*. The group observed that the infectivity levels in primary activated T cells, normally susceptible to HIV infection, dropped almost 100% when pre-treated with VacA. The block was determined to be post-reverse transcription, but pre-integration, possibly at the level of nuclear membrane. VacA was not affecting TCR signaling, but was shown to downregulate IL-2 production and secretion, leading to abrogation of proliferation, an effect similar to rapamycin. However, the group is still investigating the host target(s) of this toxin.

Roland Wolkowicz from Stanford University explained the method for screening of relatively large number of random peptide libraries for resistance to HIV infection. The rationale behind random screening for antiviral compounds was found in a possible steric block between the viral and host proteins involved in HIV lifecycle, that could lead to the gain of resistance of the cells transduced with retroviral vector carrying the peptide library formed *in silico*. Using this approach, Wolkowicz and his collaborators confirmed the positive role of the signalosome and Casein Kinase II in HIV lifecycle, as a randomly chosen peptide could interact with these proteins and block the HIV replication.

### VPU and HIV Transinfection

The first two talks in this session explored the function of HIV accessory protein Vpu. While Vpu has been well characterized to enhance virus release, the mechanism by which Vpu accomplishes this has remained unknown. Edward Stephens from the University of Kansas and his lab investigated the role of the transmembrane (TM) domain of Vpu as well as its ion channel properties by exchanging this domain for the M2 protein domain from influenza A [15]. While this exchange had little effect on replication, viral maturation or pathogenicity, the mutant virus now became susceptible to antiviral drugs that specifically targeted the M2 ion channel, namely amantadine and rimantadine. Studies of the M2 protein mapped the ion channel's function to its HxxxW motif. By replacing a single alanine residue with histidine, this group was able to construct a Vpu protein, which possessed this HxxxW motif within its TM domain. This alteration was sufficient to render HIV susceptible to rimantadine. These studies suggest the Vpu ion channel may be an effective target for anti HIV therapeutics.

Beth Noble from Paula Cannon's lab at Childrens Hospital Los Angeles presented work on the involvement of the cytoplasmic tail of Vpu in to enhancing viral release. Microscopic analysis revealed Vpu in a mutant HeLa cell line (HeLa-T17) was aberrantly concentrated in the perinuclear region; a phenotype which the group hypothesized was the result of improper trafficking with adaptor protein 3 (AP-3). AP-3 depletion by siRNA and alteration of a specific motif within the cytoplasmic tail of Vpu seem to support this hypothesis. Together, these studies have identified two specific regions of Vpu that affect viral release, namely the transmembrane ion channel and the AP-3 interacting cytoplasmic tail.

Sheila Barry from Thomas Hope's lab at Northwestern University began the discussion of HIV transinfection, by describing recent studies investigating the role of Langerhans cells (LCs) in mediating transinfection. Previously, considerable efforts have been invested in studying the effect of DC-SIGN-expressing dendritic cells (DCs) on HIV infection. Such DCs are confined to deep tissue layers, where they may not readily encounter HIV. In contrast, Langerhans cells reside in surface epithelial tissue, and can send dendritic processes across intact tight junctions to sample pathogens prior to host infection. Using a luciferase reporter assay, this group demonstrated that LCs exposed to X4-tropic virus could enhance viral infectivity in a manner similar to mature DCs. In addition, fluorescent microscopy revealed GFP-labeled HIV was found in CD1a^+ ^compartments within activated LCs and this overlap continued in recipient T cells. These results suggest LCs can enhance HIV infectivity without becoming infected themselves, and viral delivery potentially takes place through an infectious synapse resulting in delivery of both virus and LC specific proteins to target cells.

In his second talk of the conference, Derya Unutmaz presented evidence that antimicrobial peptides derived from amphibian skin (A-AMPs) inhibit both HIV infection and viral transfer between DCs and T cells [16]. Using GFP labeled virus, they observed three A-AMPs, caerin 1.1, caerin 1.9 and maculatin 1.1, could inhibit HIV infection of target cells within minutes of exposure at concentrations that did not affect cell viability. Further, caerin 1.9 could inhibit both HIV and MLV regardless of Env, while it had no effect on the non-enveloped reovirus. As DCs have previously been shown to mediate HIV transinfection by internalizing the virus and protecting viral particles from intracellular degradation, Unutmaz and colleagues considered A-AMPs might affect viral transfer from DCs to T cells. Addition of A-AMPs to HIV-pulsed DCs up to 8 hours post virus exposure was found to inhibit DC-mediated transinfection of T cells, however pretreating DCs with peptides prior to virus exposure had no effect on viral infectivity. A-AMPs were either neutralizing virus at the cell surface or trafficking to the same intracellular compartment as HIV and inactivating virus there. Through fluorescent microscopy, the group observed A-AMPs neutralized GFP labeled HIV and were confined to the surface of DCs. This suggests internalized virus may be continually cycling to the surface.

### Retrovirus Pathogenesis

Maribeth Eiden from the NIH discussed her lab's efforts in tracking the evolution of gammaretroviruses in gibbon apes and koalas. Gibbon ape leukemia virus (GALV) was originally identified in captive gibbon apes in the 1970s. Recently, koala retrovirus (KoRV) was isolated from captive koalas. Interestingly, KoRV shares a 78% nucleotide identity with GALV, despite the fact that GALV is an exogenous retrovirus affecting gibbon apes while KoRV is endogenously found in koalas. This suggests GALV and KoRV probably originated from a common ancestor, with KoRV diverging at an earlier time point than GALV. One potential source could be an infectious murine gammaretrovirus, as elements related to the envelope genes of GALV and KoRV were found in the genomes of several Asian feral mice species. In an attempt to identify potential vectors for transmission between koalas in Australia and gibbon apes in Thailand, Eiden et al found both GALV and KoRV were able to infect mosquito cells, thus establishing the possibility that insects could have acted as an infectious intermediate.

Stacey Hull from Hung Fan's lab at the University of California-Irvine closed the conference by presenting her work on the cytoplasmic tail of Jaagsiekte sheep retrovirus (JSRV) Env mediating transformation. They found JSRV-mediated transformation transpired by signaling through either the PI-3K-Akt-mTOR or the Ras-MEK-MAPK pathways, transformation was negatively regulated by p38 signaling, and phosphatidylinositol 3-kinase (PI3-K) binding site (YxxM) found in the cytoplasmic tail was necessary for transformation [17]. By conducting an alanine scan across the full length of the cytoplasmic tail, they created mutant Env proteins that affected transformation efficiency. Interestingly, one mutant increased JSRV transformation efficiency, although it was unaffected by inhibitors of the mTOR or Ras pathways, implying signaling in this mutant may be taking place through another unknown pathway. To further investigate the importance of the PI3-K binding motif, Hull exchanged the methionine residue either to aspartic acid, lysine, serine, or isoleucine. Interestingly, the isoleucine mutant, which has essentially been transformed into the binding motif for Src, had a greater transformation efficiency as compared to wildtype, thus suggesting Src may play some role in JSRV transformation.

## Competing interests

The author(s) declare that they have no competing interests.

## Authors' contributions

Every author meets the criteria of author as defined by the Retrovirology journal. SMB and MM contributed equally to the drafting and revising of the manuscript. PG and TJH also made considerable intellectual contributions to this review. All authors approved of this version for publication.
